# Нейроэндокринные особенности патогенеза синдрома поликистозных яичников (обзор литературы)

**DOI:** 10.14341/probl13350

**Published:** 2023-11-12

**Authors:** Ю. С. Абсатарова, Ю. С. Евсеева, Е. Н. Андреева

**Affiliations:** Национальный медицинский исследовательский центр эндокринологии; Национальный медицинский исследовательский центр эндокринологии; Национальный медицинский исследовательский центр эндокринологии; Московский государственный медико-стоматологический университет имени А.И. Евдокимова

**Keywords:** синдром поликистозных яичников, гонадотропин-рилизинг-гормон, кисспептин, АМГ, гипоталамус, орексины

## Abstract

Синдром поликистозных яичников (СПЯ) — одна из самых актуальных проблем в эндокринной гинекологии. Основными признаками заболевания являются гиперандрогения, менструальная и/или овуляторная дисфункция и поликистозное строение яичников по данным ультразвукового исследования. Женщины с СПЯ находятся в группе риска развития метаболического синдрома, сахарного диабета 2 типа, сердечно-сосудистых заболеваний и рака эндометрия. В связи с чем непрерывно изучаются патогенетические механизмы возникновения этого синдрома и ведется поиск новых способов лечения. СПЯ характеризуется широким спектром различных нарушений нейроэндокринной регуляции репродуктивной системы. Основной акцент обзора направлен на обобщение информации об этиологической роли нейропептидов и нейротрансмиттеров, таких как фениксин, галанины, орексины, ГАМК, в патофизиологии СПЯ и о возможности их применения в диагностических и лечебных целях. В последние десятилетия интерес ученых сосредоточен на изучении KNDy-нейронов, ведь именно синтезируемый ими кисспептин является одним из главных регуляторов гипоталамо-гипофизарно-яичниковой оси. В настоящей статье рассмотрены данные о значении KNDy-нейронов в патогенезе синдрома. Приводятся сведения о воздействии повышенных уровней андрогенов и антимюллерова гормона на ГнРГ-нейроны. Также проанализированы исследования, посвященные функциональным и структурным нарушениям в гипоталамусе при СПЯ. Поиск литературы проводили в отечественных (eLibrary, CyberLeninka.ru) и международных (PubMed, Cochrane Library) базах данных на русском и английском языках. Преимущественным являлся свободный доступ к полному тексту статей. Выбор источников был приоритетен периодом с 2018 по 2023 гг. Однако с учетом недостаточной изученности выбранной темы выбор источников датировался с 1998 г.

## Abstract (en)

Polycystic ovary syndrome (PCOS) is one of the most pressing problems in endocrine gynecology. The main signs of the disease are hyperandrogenism, menstrual and/or ovulatory dysfunction, and polycystic ovarian structure according to ultrasound. Women with PCOS are at risk for developing metabolic syndrome, type 2 diabetes, cardiovascular disease, and endometrial cancer. In this connection, the pathogenetic mechanisms of the occurrence of this syndrome are continuously studied and new methods of treatment are being sought. PCOS is characterized by a wide range of various disorders of the neuroendocrine regulation of the reproductive system. The main focus of the review is aimed at summarizing information about the etiological role of neuropeptides and neurotransmitters, such as phoenixin, galanins, orexins, GABA, in the pathophysiology of PCOS and about the possibility of their use for diagnostic and therapeutic purposes. In recent decades, the interest of scientists has been focused on the study of KNDy neurons, because it is the kisspeptin synthesized by them that is one of the main regulators of the hypothalamic-pituitary-ovarian axis. This article discusses data on the significance of KNDy neurons in the pathogenesis of the syndrome. Information is provided on the effect of elevated levels of androgens and anti-Müllerian hormone on GnRH neurons. Also analyzed are studies on functional and structural disorders in the hypothalamus in PCOS. Literature search was carried out in national (eLibrary, CyberLeninka.ru) and international (PubMed, Cochrane Library) databases in Russian and English. The priority was free access to the full text of articles. The choice of sources was prioritized for the period from 2018 to 2023.However, taking into account the insufficient knowledge of the chosen topic, the choice of sources dates back to 1998.

## ВВЕДЕНИЕ

Синдром поликистозных яичников (СПЯ) — наиболее распространенное эндокринное заболевание у женщин репродуктивного возраста, обусловленное как генетическими, так и эпигенетическими факторами, и наиболее частая причина гиперандрогении в популяции. СПЯ имеет комплекс репродуктивных, метаболических и психологических особенностей [1]. В общей популяции его распространенность составляет от 8 до 21%, и эти показатели зависят от особенностей выборки [2]. Клиническая картина, диагностика, лечение заболевания различны в зависимости от периода жизни женщины. По данным различных исследований, у пациенток с нарушениями менструального цикла частота выявления синдрома колеблется от 17,4 до 52%. У больных с клиническими проявлениями гиперандрогении (акне, гирсутизм, алопеция) СПЯ занимает ведущее место, а у женщин с ановуляторным бесплодием его выявляют в 55–91% случаев. Синдром характеризуется овуляторной дисфункцией, гиперандрогенией, поликистозной структурой яичников по данным УЗИ и различными метаболическими нарушениями, такими как инсулинорезистентность, нарушения углеводного обмена, дислипидемия, что, в свою очередь, приводит к повышенному риску развития сахарного диабета 2 типа, сердечно-сосудистых заболеваний и гиперпластических процессов эндометрия [3].

## ОСОБЕННОСТИ НЕЙРОЭНДОКРИННОЙ РЕГУЛЯЦИИ ПРИ СПЯ

В патогенезе СПЯ особую роль играют нарушения нейроэндокринной регуляции. Повышенная частота пульсовых выбросов гонадотропин-рилизинг-гормона (ГнРГ) становится ключевым фактором в развитии заболевания. При этом избыточная гипоталамическая пульсация ГнРГ приводит к преимущественной секреции лютеинизирующего гормона (ЛГ) гипофизом, что, в свою очередь, способствует овариальной гиперандрогении и овуляторной дисфункции. Частота пульсовых выбросов ЛГ у женщин с СПЯ повышена примерно на 40% по сравнению со здоровыми, что приводит к высокому уровню ЛГ в крови и относительному дефициту фолликулостимулирующего гормона (ФСГ) [4]. Гиперандрогения нарушает цепочку отрицательной обратной связи со стороны половых гормонов, и, как следствие, пульсация ГнРГ становится неконтролируемо повышенной, тем самым стимулируется избыточная секреция ЛГ, а далее — андрогенов, формируя порочный круг. Отрицательная обратная связь, опосредованная половыми стероидами, реализуется на уровень выше ГнРГ-нейронов, т.е. нейронов, секретирующих кисспептин, нейрокинин В и динорфин (KNDy-нейроны), участвующих в регуляции импульсной активности ГнРГ. KNDy-нейроны образуют обширную переплетенную сеть в аркуатном ядре гипоталамуса. Аксоны KNDy-нейронов направляются к внутренней зоне срединного возвышения, где они оказываются в близком соседстве с волокнами ГнРГ-нейронов [5]. D. Panidis и соавт. были первыми, кто сравнил уровни кисспептина у пациенток с СПЯ и здоровых женщин. Они обнаружили, что уровни кисспептина в сыворотке крови отрицательно коррелируют с индексом массы тела (ИМТ), индексом свободных андрогенов и индексом инсулинорезистентности (HOMA-IR). В других работах была зарегистрирована повышенная концентрация кисспептина при СПЯ. J. Liu и соавт. на основе большого метаанализа (1282 участницы: 699 пациенток с СПЯ и 583 женщин контрольной группы), показавшего, что уровни этого нейропептида были выше в основной группе по сравнению с контрольной, предложили гипотезу, согласно которой высокий уровень кисспептина можно рассматривать как независимый маркер синдрома [6].

Секреция ГнРГ регулируется рядом нервных и эндокринных факторов. Нейрокинин В (NKB) является одним из ключевых участников этого процесса [7]. У людей NKB кодируется геном TAC3 и преимущественно связывается с рецептором нейрокинина 3 (NK3R). NKB считается основным регулятором секреции ГнРГ. Недавние исследования показали, что путь NKB-кисспептин-ГнРГ имеет решающее значение в регуляции выброса ЛГ. Было обнаружено, что пациенты с генетически нарушенной передачей сигналов NKB имеют более низкую секрецию и частоту импульсов ЛГ. Был сделан вывод, что фармакологическая блокада NKB может стабилизировать секрецию гонадотропина и, как следствие, купировать гиперандрогению. Тем не менее необходимы дальнейшие исследования для определения точного механизма нейрокининового пути и его вклада в развитие СПЯ [6].

Гиперандрогения является ключевым компонентом СПЯ. Приблизительно 60–80% больных имеют биохимические, а 60% — клинические признаки избытка андрогенов, такие как гирсутизм, акне и алопеция [7]. Гиперандрогения негативно влияет на функцию яичников, реализуя свои эффекты через андрогеновые рецепторы. Рядом автором была зарегистрирована их чрезмерная активность в гипоталамусе, яичниках, скелетных мышцах и жировой ткани у женщин с СПЯ [8]. Растущее количество доказательств указывает на то, что андрогены участвуют в регуляции гипоталамической активности ГнРГ-нейронов [9]. Экспрессия гена KISS1 и частота импульсов ЛГ повышены у мышей с пренатальной андрогенизацией, что свидетельствует о том, что KNDy-нейроны могут быть еще одной центральной мишенью мужских половых стероидов. У мышей с нокаутом гена ароматазы наблюдается нарушение синтеза ГнРГ и снижение экспрессии гена KISS1 в переднем перивентрикулярном ядре, что приводит к дефициту эстрогенов и всплеску ЛГ [10]. Для изучения нейроэндокринного патогенетического механизма при СПЯ чаще используют экспериментальные модели животных, получавших андрогены на пренатальном этапе. Вероятно, гиперандрогения во время беременности и активированный андрогенами синтез ГнРГ у женщин с СПЯ приводят к развитию заболевания еще в эмбриональный период и далее — к манифестации в подростковом возрасте у девочки, родившейся у такой матери [11]. Клинические и фундаментальные исследования показывают, что внутриутробная среда с избыточной концентрацией мужских половых стероидов играет значительную роль в развитии данного синдрома. У дочерей, рожденных от женщин, страдающих СПЯ, увеличивается аногенитальное расстояние и усиливается функция сальных желез, что является маркером чрезмерного внутриутробного воздействия тестостерона [12]. Уровень последнего, измеренный в амниотической жидкости у больных, значительно повышен по сравнению со здоровыми женщинами в середине беременности — когда проходит наиболее критический для развития гипоталамуса период [13].

Способность трансгенно маркировать нейроны и манипулировать ими клеточно-специфичным образом на экспериментальных моделях позволяет исследователям напрямую оценивать влияние пренатального воздействия андрогенов на конкретные популяции ГнРГ-нейронов. Прямые электрофизиологические записи нейронов ГнРГ, экспрессирующих зеленый флуоресцентный белок (ГнРГ-GFP) в срезах мозга мышей, подтвердили, что их активность выше у взрослых самок мышей, получавших препараты тестостерона, по сравнению с контрольной группой [14]. Эти данные подтверждают, что андроген-опосредованные симптомы обусловлены пренатальным влиянием избыточного высвобождения ГнРГ [15].

Хорошо известно, что антимюллеров гормон (АМГ) способствует модуляции роста фолликулов яичников и в настоящее время широко используется в качестве маркера овариального резерва в клинической практике. У женщин с СПЯ уровень АМГ повышен из-за накопления мелких антральных фолликулов в яичниках [16]. С другой стороны, этот гормон может снижать экспрессию рецепторов ФСГ и ароматазы в клетках гранулезы, что ухудшает рост фолликулов и приводит к остановке их развития, формируя таким образом порочный круг. Помимо воздействия на яичники, АМГ также влияет на гипоталамус за счет высокого сродства к АМГ-рецептору AMHR2, который экспрессируется на ГнРГ-нейронах. I. Cimino и соавт. обнаружили, что этот важный гормон способен индуцировать секрецию ЛГ путем прямой стимуляции гипоталамических нейронов, секретирующих ГнРГ [17]. В исследовании B. Tata и соавт. обнаружили, что уровни АМГ в сыворотке значительно повышены у беременных женщин с СПЯ по сравнению с контрольной группой. На экспериментальных моделях (мыши), которым на пренатальном этапе вводили АМГ, были зарегистрированы значительно более высокая частота импульсов ЛГ и уровень циркулирующего тестостерона, а также более широкое аногенитальное расстояние. Авторы предположили, что терапия антагонистами ГнРГ в период гестации может редуцировать нейроэндокринные и репродуктивные нарушения у мышей. Назначение этих препаратов приводило к нормализации репродуктивной функции у взрослых мышей с пренатальным введением АМГ, что еще раз подтвердило стимулирующее действие последнего на ГнРГ-нейроны [18].

Большой интерес представляет собой исследование A.L. Barbotin и соавт., в котором изучали структуру и функцию головного мозга у женщин с СПЯ с помощью протонной магнитно-резонансной спектроскопии и диффузионно-тензорной визуализации в сочетании с трактографией волокон [19]. В дальнейшем, используя экспериментальную модель СПЯ (мыши), авторы с помощью электронной микроскопии выяснили, что повышенные уровни АМГ в сыворотке связаны с избыточной активностью гипоталамуса и аксонально-глиальной передачей сигналов, на что указывает увеличение метаболита N-ацетила-аспартата (NAA) у женщин с СПЯ. Хотя NAA в головном мозге является важным маркером функции и жизнеспособности нейронов, его вариации в содержании при магнитно-резонансной спектроскопии могут также быть связаны с осморегуляторным стрессом, жизнеспособностью нейронов или другими изменениями, которые потенциально негативно влияют на нейроэндокринные механизмы в мозге при СПЯ [20]. Зарегистрирована гипоталамическая разница концентраций NAA, наблюдаемая при данном синдроме в зависимости от его фенотипа. Кроме того, отмечено, что AMГ воздействует на микроструктурные процессы (потеря пластичности нейроглии) в срединном возвышении гипоталамуса мышей, создавая условия для секреции ГнРГ. К таким условиям относятся ретракция отростков специализированных AMГ-чувствительных эпендимоглиальных клеток, называемых таницитами, что позволяет большему количеству нейронных окончаний ГнРГ-нейронов приближаться к капиллярам срединного возвышения гипоталамуса. В этой работе, помимо различий в активности нейронов между женщинами с СПЯ и контрольной группой, также обнаружили специфическую латерализованную асимметрию в левой нижнебугорной области гипоталамуса в основной группе по сравнению со здоровыми женщинами, заключающуюся в значительно более высокой плотности волокон, пересекающих эту область. Это открытие напрямую связывает СПЯ с изменениями в соединении аркуатного ядра и срединного возвышения. Обнаружение факта пренатального воздействия избытка андрогенов на животных моделях дает убедительные доказательства того, что гиперандрогения является основным фактором патогенеза описываемой патологии, и поднимает важный вопрос о том, может ли повышение уровня мужских половых стероидов быть причиной организационных аномалий в аркуатном ядре [19].

## РОЛЬ НЕЙРОПЕПТИДОВ В РАЗВИТИИ СПЯ

Появляется все больше данных, свидетельствующих о том, что фениксин-14 и галанин влияют на прогрессирование СПЯ. Фениксин (PNX) — это недавно открытый пептид, продуцируемый в основном в гипоталамусе путем протеолитического расщепления небольшого интегрального мембранного белка. Его обнаружили в различных тканях, таких как гипоталамус, гипофиз, сердце, слизистая оболочка желудочно-кишечного тракта, островки поджелудочной железы и жировая ткань [21]. Исследования клеток передней доли гипофиза в лабораторных условиях показали, что PNX может усиливать секрецию гонадотропинов ФСГ и ЛГ путем модуляции экспрессии рецептора ГнРГ [22]. Этот пептид стимулирует развитие фолликулов, ускоряя пролиферацию клеток гранулезы человека и индуцируя секрецию эстрадиола. В эксперименте на крысах с СПЯ наблюдали повышение уровня PNX в сыворотке крови, что подтверждает предыдущие данные клинических исследований: концентрация PNX-14 была значительно выше у пациенток с СПЯ, чем у женщин контрольной группы. Интересно отметить, что концентрация пептида напрямую коррелировала с уровнями тестостерона и ЛГ и отрицательно — с уровнем эстрадиола. Считается, что повышенная экспрессия PNX-14 у пациенток с данной нозологией связана с усилением продукции ЛГ и андрогенов [23].

Одним из нейропептидов, контролирующих работу гипоталамо-гипофизарно-яичниковой оси и потенциально участвующих в развитии СПЯ, является галанин, обнаруженный в 1983 г. в центральной и периферической нервной системе. Семейство галанинов — это плейотропная группа нейропептидов с широким спектром распределения в нервной системе. Было установлено, что другие члены семейства галанинов, такие как белок, ассоциированный с передачей сообщений галанину (GMAP), галаниноподобный пептид (GALP), аларин и спексин, эволюционировали посредством серии дупликаций генов из общего предкового пептида [24]. Галанин вырабатывается в гипоталамусе и передней доле гипофиза, где его синтез активно стимулируется эстрогенами. Данный нейропептид контролирует секрецию ГнРГ и предовуляторные пики ЛГ, а также регулирует стероидогенез в ткани яичника. F. Azin и соавт. исследовали роль галанина в развитии СПЯ на экспериментальной модели (крысы). Внутрибрюшинная инъекция пептида вызывала повышение уровня ФСГ, снижение ЛГ и инсулина, тем самым нивелируя метаболические нарушения у животных [25]. S.O. Altinkaya сравнил уровни галанина в сыворотке у 44 пациенток с СПЯ и у 44 здоровых женщин того же возраста и обнаружил более низкие уровни этого пептида в основной группе. Лечение галанином может стать перспективным терапевтическим подходом при овариальной гиперандрогении с учетом его метаболической активности [26].

Еще одним биоактивным пептидом семейства галанинов является аларин, состоящий из 25 аминокислот и открытый в 2006 г. Первоначально R. Santic и его коллеги обнаружили аларин в ганглиоцитах нейробластических опухолей человека [27]. Широкое распространение пептида подчеркивает его разнообразный спектр физиологических функций, а экспрессия в головном мозге связана с центральной регуляторной ролью в пищевом поведении, энергетическом гомеостазе, поддержании температуры тела и секреции половых гормонов [28]. Кроме того, его скопление вокруг кровеносных сосудов кожи и глаз обеспечивает вазоактивный, противовоспалительный, противоотечный и противомикробный эффекты. Также показано, что аларин участвует в развитии различных патологических состояниях, таких как ожирение, сахарный диабет 2 типа, диабетическая ретинопатия, СПЯ и депрессия [29]. На экспериментальных моделях было продемонстрировано, что аларин, подобно другим представителям семейства пептидов галанина, регулирует активность гипоталамо-гипофизарно-гонадной оси у крыс и активирует секрецию ГнРГ и ЛГ, как следствие, стимулирует синтез половых стероидов [30]. В клинических исследованиях получены результаты, которые свидетельствуют о значимом повышении уровня этого пептида у пациенток с СПЯ по сравнению с контрольной группой [31][32]. Исследование U. Gorkem и E. Yildirim было первым, подтвердившим связь между уровнями аларина в сыворотке крови и СПЯ: у больных наблюдалась повышенная концентрация данного пептида и положительная корреляция между его уровнем и показателем ЛГ. Это же исследование продемонстрировало, что высокое значение аларина повышает риск развития СПЯ, на основании чего авторы предлагают считать этот пептид независимым предиктором заболевания [32]. Более того, в работе M.Q. Li и соавт. уровень аларина положительно коррелировал с такими показателями, как ИМТ, ЛГ и уровень андрогенов. Наличие инсулинорезистентности сопровождалось повышением концентрации этого пептида у женщин с СПЯ по сравнению с женщинами без нее, что делает его еще и метаболическим маркером [33].

Особый интерес представляют исследования роли орексинов в области эндокринной гинекологии. На сегодняшний день они затрагивают такие проблемы как связь с СПЯ, функциональной гипоталамической аменореей, эндометриозом, нарушениями сна. В 1998 г. две исследовательские группы независимо друг от друга открыли новую систему гипоталамических нейропептидов, назвав их орексинами/гипокретинами [34][35]. Гипокретиновая система состоит из двух пептидов, гипокретина-1 и гипокретина-2 (HCRT1 и HCRT2; также называемых орексин-А и орексин-В), которые действуют через два G-связанных рецептора: HCRTR1 и HCRTR2 [36]. HCRTR1 обладает большим сродством к орексину-А, тогда как HCRTR2 неселективен и связывает орексин-А и орексин-В с равной аффинностью [34]. Гипокретинсодержащие нейроны расположены в заднем и латеральном гипоталамусе и имеют широко распространенные проекции по всему головному и спинному мозгу [37]. Установлено, что орексины принимают участие в модулировании функций нейроэндокринной системы, инициируя прием пищи, активизируя моторную активность и энергетический обмен, провоцируя состояние бодрствования и угнетая обе фазы сна, регулируя циркадные изменения температуры тела. Также показано, что орексин-содержащие нейроны гипоталамуса могут повышать активность симпатической нервной системы, и участвовать в регуляции ответных реакций на стресс [38]. Стало известно об экспрессии орексина-А в некоторых периферических и эндокринных тканях, таких как надпочечники, гипофиз, яичники и яички [37]. Установлена взаимосвязь между данными нейропептидами и гипоталамо-гипофизарно-яичниковой системой [39].

Хотя физиологическая роль орексиновой системы точно не известна, имеются публикации, касающиеся регуляторной функции орексина-А в системе питания [40]. Исследования показали, что он подавляет секрецию инсулина и увеличивает высвобождение глюкагона [41], в то время как другие работы in vivo и in vitro выявили его противоположные эффекты [42]. На экспериментальных моделях было установлено, что при инсулинорезистентности снижалась концентрация данного пептида, а введение агонистов рецепторов орексина-А восстанавливало чувствительность тканей к инсулину. Это позволяет выдвинуть гипотезу о том, что концентрация этой молекулы будет служить более надежным показателем наличия инсулинорезистентности, чем расчетные индексы НОМА-IR и Сaro при СПЯ [43]. В подтверждение этой гипотезы E. Yilmaz и соавт. обнаружили более низкий уровень орексина-А в сыворотке у пациенток с СПЯ по сравнению с контрольной группой, а также его отрицательную корреляцию с систолическим артериальным давлением, гирсутным числом по шкале Ферримана–Голлвэя, уровнями ЛГ и свободного тестостерона [44]. Потенциально приводить к снижению концентрации изучаемого пептида в сыворотке крови у женщин с СПЯ может и гиперандрогения. Пока механизмы, регулирующие синтез и секрецию орексина-А при СПЯ, неизвестны, поэтому требуются дальнейшие исследования, которые ответят на вопрос, является ли снижение уровня орексина-А, наблюдаемое при СПЯ, опосредованным инсулинорезистентностью или результатом других метаболических процессов.

## НЕЙРОТРАНСМИТТЕРЫ: ЗНАЧЕНИЕ В ПАТОГЕНЕЗЕ СПЯ

В этиологии СПЯ важно обратить внимание на структуру и функциональность основного профиля нейротрансмиттеров. Как упоминалось ранее, нарушение пульсирующей секреции ГнРГ и регуляции гипоталамо-гипофизарно-яичниковой оси считаются ключевыми особенностями, лежащими в основе патофизиологии этого синдрома. Нейротрансмиттеры, такие как ГАМК (гамма-аминомасляная кислота), глутамат, серотонин, дофамин и ацетилхолин, а также опиоидная система, могут препятствовать нормальной секреции ГнРГ [45].

ГАМК является основным тормозным нейротрансмиттером в центральной нервной системе. M.S. Silva и соавт. доказали, что ГАМК-нейроны аркуатного ядра стимулируют секрецию ГнРГ и приводят к мощному ответу ЛГ [46]. В аналогичных работах было обнаружено, что женщины с СПЯ имели повышенный уровень ГАМК в спинномозговой жидкости по сравнению с участницами с регулярным менструальным циклом [47]. В работе D.T. Porter и соавт. сообщается, что количество ГАМКергических синапсов на KNDy-нейронах значительно увеличилось у овец, подвергшихся воздействию тестостерона на пренатальном этапе, следовательно, ГАМК может активировать KNDy-нейроны, а также ГнРГ-нейроны, повышая частоту импульсов ГнРГ и ЛГ при СПЯ [48]. Также хроническая активация ГАМК-нейронов вызывает у мышей развитие клинических признаков, характерных для определенных фенотипов этого синдрома: гиперандрогению, нерегулярный эстральный цикл и олиго/ановуляцию. Кроме того, на гипоталамических ГАМК-нейронах обнаружена меньшая экспрессия рецепторов к прогестерону у мышей с СПЯ, что ослабляет ГАМК-опосредованный эффект обратной связи прогестерона на ГнРГ-нейроны [49].

Роль глутамата как основного возбуждающего нейротрансмиттера в ЦНС в патогенезе СПЯ до сих пор неясна. Нейроны ГнРГ экспрессируют как ионотропные (AMPA, KAR, NMDA), так и метаботропные глутаматные рецепторы, однако работ, описывающих роль последних в регуляции ГнРГ, мало [50]. Антагонист NMDA-рецепторов MK801 блокировал эндогенные импульсы секреции ГнРГ, тогда как пульсирующее высвобождение гормона не нарушалось в присутствии 6,7-динитрохиноксалин-2,3-диона (блокатор каинатных рецепторов) [51]. На мышиной модели СПЯ, индуцированной пренатальной андрогенизацией, влияния глутамата на пульсацию ГнРГ не установлено [49]. J.F. Kawwass и соавт. обнаружили повышенные уровни этого нейротрансмиттера в спинномозговой жидкости больных описываемой патологией по сравнению с контрольной группой [47]. Исследования на животных позволили выявить избыток глутамата и высокую экспрессию рецептора N-метил-D-аспартата у крыс с индуцированным СПЯ, что предполагает прямую избыточную стимуляцию высвобождения ГнРГ и ЛГ [52]. В работе N. Chaudhari и соавт. оценивали статус нейротрансмиттеров путем определения их уровней, метаболизма и экспрессии рецепторов в гипоталамусе, гипофизе, гиппокампе и лобной коре головного мозга на экспериментальной модели (крысы). Уровень серотонина был значимо снижен во всех проанализированных тканях с минимальными показателями в гипоталамусе и гипофизе в основной группе по сравнению с контролем. Аналогичная тенденция наблюдалась в отношении содержания норэпинефрина. Уровни адреналина были снижены в гипоталамусе и гипофизе животных с СПЯ, но в гиппокампе и лобной коре не наблюдалось различий. Кроме того, во всех протестированных тканях головного мозга основной группы наблюдались заметно низкие уровни дофамина и ГАМК по сравнению с контролем. В отличие от вышеперечисленных нейротрансмиттеров количество глутамата было значительно повышено в гипоталамусе, гипофизе, гиппокампе и лобной коре крыс основной группы [51].

Регулирующим эффектом в отношении репродуктивной функции обладают также пептиды опиоидной системы, которая включает эндорфины, энкефалины и динорфины. Эндорфины отвечают за обезболивание, реакцию на стресс и эмоциональные процессы, а также репродуктивную нейроэндокринную функцию. M. Kiałka и соавт. в своем исследовании наблюдали повышенный уровень эндорфинов в сыворотке у пациенток с СПЯ по сравнению с контрольной группой, эти данные также коррелировали с уровнями инсулина и глобулина, связывающего половые гормоны. Поскольку известно, что ожирение само по себе влияет на уровень эндорфинов, в исследование были включены только женщины с нормальным ИМТ [53].

О роли ацетилхолина — основного медиатора парасимпатической нервной системы — в патогенезе СПЯ известно немного. Парасимпатическая иннервация яичников осуществляется блуждающим нервом. На экспериментальных моделях (крысы с СПЯ, индуцированным инъекцией эстрадиола валерата) односторонняя или двусторонняя перерезка блуждающего нерва восстанавливала спонтанную овуляцию в обоих яичниках у 75% объектов [54]. В другой работе животным (крысам) вводили 700 мг атропина в качестве конкурентного антагониста мускариновых ацетилхолиновых рецепторов, что привело к спонтанной овуляции более чем в 70% случаев [55]. Интерес для исследователей представляет потенциальный регулирующий эффект ацетилхолина на выброс ГнРГ. В культивируемой клеточной линии GT1–7 данный медиатор стимулирует высвобождение ГнРГ через активацию никотинового рецептора, в то время как ингибирующий эффект на активность ГнРГ был опосредован активацией мускаринового рецептора [56]. Воздействие экзогенного медиатора на культивируемые клетки передней доли гипофиза приводило к снижению реакции на ГнРГ-индуцированное высвобождение ЛГ. Этому ответу противодействовал антагонист мускариновых рецепторов атропин [57]. В исследовании N. Chaudhari и соавт. активность ацетилхолинэстеразы, гидролитического фермента ацетилхолина, была повышенной в гипоталамусе и гипофизе крыс с СПЯ наряду со сниженной экспрессией мускаринового рецептора ацетилхолина, что позволяет предположить снижение его уровня при развитии заболевания, а это, в свою очередь, приведет к увеличению пульсационных выбросов ГнРГ и ЛГ [51].

Интересны работы, в которых изучалась роль катехоламинов в регуляции ГнРГ. Было показано, что норэпинефрин отвечает за предовуляторный выброс ЛГ через α- и β-адренергические рецепторы. Пропранолол, блокатор α-адренергических рецепторов, стимулирует норэпинефрин-индуцированное высвобождение ЛГ, в то время как лечение β-антагонистом блокировало пик ЛГ. Вероятно, стимулирующее действие норэпинефрина на высвобождение гормона опосредуется β-адренергическими рецепторами, в то время как α-адренергические рецепторы ингибируют его высвобождение. Исследований, оценивающих регуляторный в отношении ГнРГ эффект адреналина, немного, но ученые предполагают наличие стимулирующего влияния через α-адренергический рецептор [58].

В отличие от норадреналина и адреналина, дофамин является основным супрессором высвобождения ГнРГ [59]. Была высказана гипотеза о наличии связи между СПЯ, низким уровнем дофамина и гиперпролактинемией [60]. Известно, что последняя оказывает ингибирующее действие на гонадотропины [59]. В исследовании N. Chaudhari и соавт. содержание дофамина в головном мозге крыс с СПЯ было значительно снижено наряду со сниженной экспрессией D2-рецепторов дофамина, что может привести к гиперсекреции пролактина [51]. Эти работы подтверждают значимую роль медиаторов симпатической и парасимпатической систем в патогенезе развития СПЯ.

## ЗАКЛЮЧЕНИЕ

СПЯ — мультифакторное заболевание женщин репродуктивного возраста, патофизиология которого до конца не изучена. Недавние достижения в области нейроэндокринологии значительно продвинули наше понимание механизмов, лежащих в основе развития синдрома. Аномальная активация ГнРГ-нейронов гипоталамуса и избыточный синтез андрогенов в яичниках являются важнейшими патогенетическими звеньями при СПЯ. Появляется все больше работ, изучающих влияние нейропептидов, таких как фениксин, галанины, орексины на синтез и секрецию ГнРГ, что позволит в дальнейшем разработать методики их применения в диагностических целях. Большой интерес вызывают не только исследования функциональных нарушений гипоталамо-гипофизарной оси, но и структурных особенностей в областях, ответственных за синтез и секрецию ГнРГ при СПЯ. Все это позволяет нам считать именно головной мозг главным дирижером в развитии синдрома. СПЯ является одной из самых актуальных проблем в эндокринной гинекологии, и новые экспериментальные данные о патогенезе станут основой эффективных схем диагностики и лечения в ближайшем будущем.

## ДОПОЛНИТЕЛЬНАЯ ИНФОРМАЦИЯ

Источники финансирования. Работа выполнена в рамках государственного задания №123021300169-4 «Эпигенетические предикторы и метаболомная составляющая аменореи различного генеза у женщин репродуктивного возраста», 2023–2025 гг.

Конфликт интересов. Авторы декларируют отсутствие явных и потенциальных конфликтов интересов, связанных с содержанием настоящей статьи.

Участие авторов. Все авторы одобрили финальную версию статьи перед публикацией, выразили согласие нести ответственность за все аспекты работы, подразумевающую надлежащее изучение и решение вопросов, связанных с точностью или добросовестностью любой части работы.
